# Effectiveness of Method of Repair of Incidental Thoracic and Lumbar Durotomies: A Comparison of Direct Versus Indirect Repair

**DOI:** 10.7759/cureus.5224

**Published:** 2019-07-24

**Authors:** James Brazdzionis, John Ogunlade, Christopher Elia, Margaret Rose Wacker, Rosalinda Menoni, Dan E Miulli

**Affiliations:** 1 Neurosurgery, Riverside University Health System Medical Center, Moreno Valley, USA; 2 Neurosurgery, Arrowhead Regional Medical Center, Colton, USA

**Keywords:** durotomy, incidental durotomy, csf fistula, pseudomeningocele, repair, csf leak

## Abstract

Introduction

Incidental durotomy (ID) is a well-known complication in spine surgery. Surveys have not identified a consensus for repair method among neurosurgeons. IDs may lead to complications such as cerebrospinal fluid (CSF) fistula, which may predispose patients to infection, additional procedures, increased length of stay and morbidity. This study aims to compare durotomy repair methods with clinical outcomes.

Methods

The neurosurgery database at a single institution, Arrowhead Regional Medical Center, was screened for all patients who underwent thoracic and lumbar spine surgery from 2007-2017. Retrospective chart review of operative reports identified patients with an ID. Data collection included: length of stay, infection, additional procedures, time lying flat, CSF fistula formation (primary endpoint) with analysis using t-tests.

Results

A total of 384 patients underwent initial analysis. Of the 384 patients, 25 had an incidental durotomy based on operative reports. Four patients were excluded from this subset: two were repaired with muscle graft (low N), two were excluded for unclear repair method. The remaining 21 were stratified into two groups, those repaired directly with suture with or without adjunct (N=9) and those repaired indirectly with sealant (N=12). No patients developed a CSF fistula. The indirect group had a length of stay of six days, while the direct group had a length of stay of four days, p=0.184. Two of the nine patients in the direct group and two of the twelve patients in the indirect group developed an infection, p=0.586.

Conclusion

No patients developed CSF fistulas. Secondary endpoints of length of stay and infection rate did not differ. This study was unable to determine if direct versus indirect repair was a more effective repair method for ID. It is possible that if an incidental durotomy is identified and repaired with a water-tight seal, the repair method does not affect the outcome. It is up to the surgeon to individualize repair based on ability and circumstances.

## Introduction

Incidental durotomy (ID) is a well-known complication of spinal surgeries with some studies noting a baseline incidence of 3.5% [1]. Standardized repair for large and pinhole durotomies has not been identified. Repair methods vary between surgeons in terms of utilization of primary repair, fibrin glue, fibrin coated fleeces as well as muscle graft [2-4].

Numerous technical papers and publications have identified individualized success of several repair mechanisms for specific situations such as subarachnoid pleural fistulas, arachnoid cysts, ventral repair techniques, muscle graft and fibrin glue usage in minimally invasive surgery (MIS), clips in MIS lumbar spine surgeries, and transthecal repair for ventral cerebrospinal fluid (CSF) durotomies [5-15]. However, studies comparing the effectiveness of all repair methods are limited and data is mixed with respect to outcome. Some studies have found successful results using indirect methods such as gel-foam, polyethylene glycol (PEG) grafts or hydrogels, and autologous blood patch [16-18]. Additional studies have attempted to define systematic approaches to repair but without emphasis on the selection of the method of repair [19]. Thus, the literature supporting individualized methods and techniques is vast but a defined consensus for the treatment of incidental durotomies is yet to be discovered.

Results have varied in the determination of the impact of long-term outcomes of these dural tears, however, it is suggested that patients, especially those who require re-operation, may have worse long-term outcomes in pain relief, incur longer lengths of stay, and have increased rates of infection [20, 21]. If we can reduce these risks by ensuring an appropriate watertight seal to minimize complications, it may be possible to improve these outcomes. Thus, the goal of this paper was to identify if the method of repair, either direct with suture (with or without an adjunct such as fibrin glue) or indirect repair (with an alternative method such as fibrin glue, or PEG hydrogels), differed in regards to developing significant complications of CSF fistula or other secondary outcome measures.

## Materials and methods

The internal neurosurgical database of a single institution, Arrowhead Regional Medical Center, was reviewed retrospectively. Data of all patients undergoing thoracic or lumbar spine surgery from 2007 to 2017, were collected using inpatient and post-discharge records. Initial inclusion criteria for analysis included all patients on the neurosurgical internal database with listed lumbar or thoracic intervention without cervical spine involvement. A retrospective review of operative notes identified all cases in which an incidental durotomy was encountered for inclusion in the study. Cases in which intentional durotomy, such as for intradural tumors, was planned and identified in the operative report were excluded from the study. Furthermore, if on an initial review of the neurosurgical database entries documented an intradural pathology such as a tumor or procedure, they were not included for initial review. Patients with cervical spine involvement of the procedure or surgical construct were excluded from the review. The primary endpoint measured was the presence of a CSF fistula measured by frank CSF drainage, development of symptoms suggestive of CSF fistula or subcutaneous fluid collection found to be a CSF fistula. Method of repair, length of time laying flat, length of stay in the hospital, infection, and need for additional procedures were collected. Patients without data on method of repair were excluded from the analysis. Patients were stratified into groups based on the method of repair; muscle graft, direct repair with suture and indirect repair with sealants such as a synthetic dural matrix or fibrin glue. Patients in the muscle graft subset (N=2) were excluded from statistical analysis for a low sample size. Data were assessed for statistical significance between groups using t-tests on IBM SPSS Statistics.

## Results

A total of 384 patients who underwent thoracic and lumbar spine intervention and met inclusion criteria for additional analysis. Incidental durotomies were observed in 25 patients. Patients were stratified into two groups: group 1 with IDs directly repaired using suture, and group 2 with IDs indirectly repaired with a sealant or clips. In each group, the repair may additionally have been supplemented with an adjunct such as an addition of fibrin glue or hemostatic agent. Four patients were excluded from the statistical analysis from the initial subset of 25. Two patients were excluded as they were repaired with a muscle graft and outcome projections would not be able to be effectively be concluded with only two subjects. One patient was excluded as the method of repair was not clearly documented in the operative report, and one final patient was excluded as the durotomy was determined to be irreparable. Group 1 contained 9 patients and group 2 contained 12 patients. Demographics of the study population are listed in Table 1. Demographics of the two groups did not vary except for the procedure performed. Patients repaired directly with suture exclusively underwent lumbar procedures compared to the indirect repair group with an equal distribution of lumbar and thoracic procedures. No patients from either group developed the primary outcome measure of symptomatic CSF fistula. Thus, no patients from either group required an additional procedure or returned to the operating room for incidental durotomy. Secondary outcomes of length of stay and infection rates were calculated for each group. In the direct repair group, the median length of stay was four days and infections were identified in two of the nine of the patients. In the indirect repair group, the length of stay was six days, with two of the 12 patients complicated with an infection. P-values for the length of stay and infections were 0.184 and 0.586 respectively and not statistically significant as calculated using t-tests (Table 2). Size, location of durotomy and duration of time lying flat were excluded from analysis as this information was variably documented in the medical records.

**Table 1:** Demographics of Study Participants Demographics data from patients undergoing direct or indirect repair are presented above. P-values for race and procedure are comparisons determining statistical significance in the respective categories of race and procedural regions as a whole between the two repair groups.

	Group 1 (Direct Repair)	Group 2 (Indirect Repair)	P-value
Age (Mean)	50	43.25	0.158
Race			0.738
Hispanic	5	5	
Caucasian	3	4	
African American	1	3	
Procedure (Region)			0.010
Thoracic	0	6	
Lumbar	9	6	

**Table 2:** Summary of Outcomes Measured by Group with P-values

	Group 1 (Direct)	Group 2 (Indirect)	P-value
Length of Stay (median)	4	6	0.184
Infection (rate)	0.222222	0.166667	0.586
Cerebrospinal Fluid Fistula (occurrences)	0/9	0/12	N/A

## Discussion

Incidental durotomies are a well-known complication from thoracic and lumbar intervention when working near dural covering of the spinal cord. Intra-operatively incidental durotomies are identified directly under visualization and may be re-assessed with a Valsalva maneuver. Durotomies may result in complications including infections and may be symptomatic with meningitis symptoms that may both prolong stay and require the patient to be exposed to additional procedures such as lumbar drains, or re-exploration and closure contributing to morbidity and mortality.

Several studies have identified different methods of primary repair of incidental durotomies. Some authors have advocated for primary repair with suture, while others have noted that hydrogel sealants or alternatives are effective as closure modality [12, 17, 18, 22]. Literature has even sought to develop a treatment approach to repair, however, despite defining an approach to treatment, selection of utilization of a graft, using suture alone or alternative method was not defined [19]. As there is no clear consensus in the literature our data sought to clarify whether one modality of closure was more effective than another in repair.

No significant difference in primary outcome measures of development of CSF fistula in either method of repair studied was identified i.e., direct primary closure with suture with or without adjunctive fibrin glue versus indirect closure with glue and synthetic analog (including clips). No patients in either group developed a symptomatic CSF fistula or symptoms consistent with meningismus. Additionally, no patients in either group required a re-operation or a secondary procedure for the above complications. No significant differences between secondary outcomes of infection rates or length of stay were identified. We note that the comparison of outcomes in these two groups has been challenging as baseline data for complication rates remain widely inconsistent. Despite this, symptomatic CSF fistulas and their significant complications including meningitis are well documented in the literature [23]. However, without strong baseline incidences, a larger sample of patients is required to capture more of these rare occurrences with primarily repaired incidental durotomies, to increase the power of the study and to draw more significant conclusions.

This data seems to imply that the method of repair does not significantly alter the outcome. Incidental durotomies are overall a rare occurrence that can occur from a variety of etiologies ranging from surgeon error to spontaneous occurrence. Although studies have reported rates of 3.5%, the true incidence may be different due to under-reporting [1]. Furthermore, complications such as CSF fistula certainly do not occur in each patient with an incidental durotomy. Our study suggests that, with newer techniques to repair these defects and improving surgeon experience, these complications are treatable and are unlikely to result in serious complications. However, large scale conclusions would require a larger sample size. Regardless of the method of repair, patients that received appropriate repair did not develop a CSF fistula and length of stay and infectious parameters did not differ between groups. Therefore, even though no patients developed the rare but significant complication of CSF fistula, secondary outcomes between the two groups did not significantly differ, which implies that overall outcome may not be affected by the method of repair. As the method of repair was not randomized and was instead selected by the surgeon, this may imply that the surgeon should examine each durotomy and determine the most effective method of repair for the individual defect. Ensuring a watertight seal to prevent CSF leak is a well-documented goal of primary repair which should be ensured regardless of the repair method. Further prospective trials with larger patient populations contributing to larger statistical power should be conducted to further investigate this.

## Conclusions

In our subset population, none of the patients who had incidental durotomies that underwent primary or indirect repair developed symptomatic CSF fistulas. Secondary endpoints of the length of stay and infection rates did not differ based on the method of repair. Therefore, this study was unable to determine any difference between the method of repair when comparing suture as a primary repair mechanism to fibrin glue with or without adjunct. This study is unable to support one method of repair (direct repair with suture versus indirect repair) over the other, however, a watertight seal is effective in preventing further long-term complications. The operating surgeon must determine the best method for the individual defect based on their resources, morphology of the defect and ability. Further prospective studies are needed in order to identify if there truly is a more effective repair mechanism for ID.
